# Earning opportunities and informal payment as influencing factors in medical students’ speciality choice

**DOI:** 10.1186/s12875-021-01608-4

**Published:** 2021-12-30

**Authors:** András Mohos, Thomas Frese, László Kolozsvári, József Rinfel, Albert Varga, Csenge Hargittay, Dalma Csatlós, Péter Torzsa

**Affiliations:** 1 Faculty of Medicine, Department Family Medicine, University of Szeged, 6725 Tisza Lajos krt. 109, Szeged, Hungary; 2 Institute of General Practice and Family Medicine, Martin-Luther-University Halle-Wittenberg, Magdeburger Straße 8, 06112 Halle/Saale, Germany; 3 Department of Family and Occupational Medicine, Faculty of Medicine, University of Debrecen, 4028 Kassai út 26, Debrecen, Hungary; 4 Institute of Primary Care, University of Pécs, 7623 Rákoczi St. 2, Pécs, Hungary; 5 Department of Family Medicine, Semmelweis University, 1085 Stáhly u. 9, Budapest, Hungary

**Keywords:** Career choice, Medical student, Income, Informal payment, Family physician, Hungary

## Abstract

**Background:**

The Hungarian primary care system faces a severe shortage of family physicians. Medical students’ perceptions of family medicine need to be known and medical students need to be given appropriate and comprehensible information about this speciality. The expected future salary is an important factor in career choice. Most of the family doctors are self-employed and the practices have a corrected capitation-type financing. Although the majority of health care services are covered by social health insurance and are provided for the insured patients free of charge, informal payment is an existing phenomenon with different motivations and consequences. This study aimed to investigate medical students’ knowledge about their future earning opportunities and their attitudes towards informal payment.

**Methods:**

A cross sectional survey with a self-administered questionnaire was conducted. Each of the four Hungarian medical universities were represented by their medical students who attended family medicine lectures in person from December 2019 to April 2020. The students were asked about their career plans, about their estimations of current and ideal expected salaries and about the effect of expected income for the choice of specialisation. Their attitudes towards informal payment were assessed.

**Results:**

Response rate was 67.3% (*N* = 465/691). Almost two-thirds of the participants were women. Only 5% of the respondents (*N* = 23/462) plan to work as a family doctor in the future. The vast majority (91.9%) of the students had already thought about their future income. On a 10-point Likert scale (1 = ‘no influence’, 10 = ‘very big influence’) 76% answered that the expected future income exerts a considerable (≥5 Likert points) influence on their career choice in general. The mean of the ideal expected monthly income of the residents, GPs and other specialists was €1154 ± 648, €1696 ± 904 and €2174 ± 1594, respectively. The mean of the monthly income for a GP, as estimated by the studenst, was €1140 in rural and €1122 in urban settings. More than four-fifths of the students (*N* = 375/453) rejected the practice of informal payment.

**Conclusions:**

Expected salaray is one important aspect in the career choice of medical students, students wish to have more information on this topic. The reported ideal incomes are higher than those expected. This points to a relevant gap. However, most of the students do not accept informal payment as a possibility to close this gap. The expected and the ideal income differ from the real incomes of Hungarian GPs – this indicates the need of bringing objectoive information to the students to enhance attractivity of GP as a carer choice.

## Background

The healthcare system requires available, accessible, acceptable and well-qualified human resources. These are key factors in effective, high quality health services [1]. Hungary faces a considerable challenge in this sector, especially in primary care. The aging and the migration of active family physicians, paired with the growing number of vacant practices points to an increasingly burning problem [2].

For successful human resource recruitment in primary care, medical students’ perceptions of family medicine and their motivations regarding career choice and specialisation need to be better understood [3]. Many factors could influence the choice of speciality. Some of them are unchangeable, for example gender, origin or family role model. On the other hand, other factors can be influenced, such as education or experiences. The expected salary or income (the two terms are used synonymously in the present paper) could also influence career plans and specialisation [4]. Career choice as a multifactorial process can be examined from many aspects and measured with many tools. In our research we asked students about these factors but in this article we focused only on the the impact of earning opportunities and informal payment on career choice [5].

When we examine the financial aspects of specialisation, we have to understand doctors’ different earning opportunities. In this survey, we are focusing on the state healthcare system only and do not include the private sector. The doctors in Hungarian secondary care mostly work as civil servants. Their monthly salary varies from approximately €470–1170 depending on work hours and level of qualification [6]. Family doctors or family physicians (the two terms are used synonymously in the present paper) work in Hungarian primary care and only few of them are civil servants. They are mostly self-employed, have their own practices and a contract with the National Health Insurance Fund of Hungary. The practices have a corrected capitation-type financing and from this remuneration the family doctor is able to calculate their employees’ and their own salary. Therefore, we cannot determine a unified salary for family physicians. Based on national financial data, we determined €1000 as the average monthly salary for a family physician in our study. In comparison to the data of Hungarian Central Statistical Office, the average monthly salary in Hungary, with the use of the central exchange rate, was €739 in April 2020 [7]. More important than the absolute value of the expected income is how it relates to the ideal expected income of students. The ideal expected income is understood as the income that provides an opportunity to ensure the adequate standard of living envisioned by the students and is suitable for expressing the social prestige of the medical profession. If we want to understand the real meaning of these incomes in an international context, we can use the concept of purchasing power parity (PPP). PPP is a macroeconomic metric to compare economic productivity and standards of living between countries through to buy the same amount of goods and services in each country. Hungary’s PPP was 31.914 US Dollars, Germany’s 52.386 US Dollars and the EU28’s 43.188 in 2018 [8].

In Hungary, the majority of health care services are covered by social health insurance and are provided for the insured patients free of charge. Participation in the social health insurance system is compulsory. Employers pay a “social tax”, whereas employees pay a social health insurance contribution. The contribution of some special groups (e.g. old age pensioners, minors and students) is covered by the government. The population of Hungary was almost completely covered (94.9%) in 2017 [9, 10]. However, informal payment, called “gratitude payment” in Hungary, is an existing practice. Patients often give money to their caregivers. The motivation is not only the expression of gratitude, but they also try to secure “advantages” for themselves. On the other hand, as a legacy of socialist times when gratitude payment was seen as a tacitly accepted supplement to extremely low official incomes, many of the doctors still count on this money. It is a particular characteristic of the former Eastern Block states, but is also present in African, South-American and some Asian healthcare systems, and it is a current practice in Turkey and Greece. This method distorts the system, as it is contrary to principles of professional work and adversely influences doctor-patient relationships. The frequency of gratitude payment varies from specialisation to specialisation, but there is a significant group of doctors for whom this illegal income supplement plays an important role. Therefore, theoretically, they are against this practice but, in their daily routine, they are constrained to accept it because of financial reasons. Although many of the doctors and a considerable part of society are against the practice of informal payments, changes on systematic and social level are required to eliminate it [11, 12].

This study had two main objectives:

1. It aimed to investigate medical students’ knowledge about their future earning opportunities in primary care and in secondary care. This, in turn, was compared with the estimated future salary and the expected ideal salary. We investigated the relationship between financial aspects and career choice, especially in terms of the specialty of family medicine.
2. We were interested in medical students’ attitudes towards informal payment.

There were two hypotheses underlying this research: If family physicians were paid closer to the income perceived as ideal by students, the problem of a severe lack of young family physicians could be reduced. If the income of those working as physicians better matched their feelings of appropriateness and justice, the problem of informal payments would be reduced. The study did not test these hypotheses directly but adds relevant information to the topic by revealing what students think of how it should be. As this was a cross-sectional study, it was not appropriate to directly investigate causal relationships, but the results obtained may help to further investigate and discuss the topic on a scientific basis.

## Methods

### Study design and participants

In our cross-sectional survey we used a self-administered paper based questionnaire. Participation was voluntary and anonymous. Each of the four Hungarian medical universities (Budapest, Debrecen, Szeged, and Pécs) were represented by their fourth and fifth year medical students who attended face-to-face family medicine lectures at that time. At the end of the lectures, the students received a xeroxed copy of the questionnaire, filled it in and handed in before leaving. Because the four universities have different curricula and family medicine is included in different years, we had to involve the appropriate years everywhere. Data collection was carried out from December 2019 to April 2020. After this time, it had to be stopped due to the COVID-19 pandemic. After receiving appropriate information about the study, 465 students decided to participate in our study. Unfortunately, because of the Covid-19 Pandemic, we reached a lower response rate than we had initially expected. However, the nature of the limiting factor we can assume that the characteristic of the participant group of the students and the non-participant group of the students are not different. The gender ratio supports our assumption: it was 62.1% (288/464) in the participant group and 63.9% (379/593) in the non-participant group.

### Questionnaire

We used a self-developed questionnaire. We collected sociodemographic data such as gender, age, place of origin, family role models (in terms of higher education attainment, medical degree or family physician in the family). Because of the heterogeneity of the definitions of rural and urban, we did not use strict categories in the categorisation of the settlements. The capital city is obviously Budapest, but when it came to the other categories (big city, small town, or rural area), the participants were free to decide their category. There were questions concerning future career plans: preferred speciality, institution and workplace. We assessed the effect of future income for the choice of specialisation: students’ previous search for information about this topic, influence of possible salary on career choice, estimation of current and ideal salaries, and students’ self-rated confidence regarding their estimations. We also asked students about their attitude towards informal payment. The scales and questions used to measure the impact of earning opportunities and informal payment have already been used in other studies in the international literature [13, 14]. The intelligibility of the questionnaire was tested by a group of medical students who do their thesis at the Department of Family Medicine in Szeged.

### Data analysis

We used IBM SPSS Statistics 24 Software for statistical analysis. Descriptive statistics were given in terms of counts and percentages, means and standard deviations (SD), respectively, complemented by medians and quartiles where appropriate. N’s vary due to missing values. The data were analysed by univariate cross tabulation. The percentages were compared by χ2 statistics. The one-way analysis of variance (ANOVA) test was used to compare means and to determine whether there are any statistically significant differences between the independent groups. For further analysis of the categories the Scheffe post hoc test was administered. Statistical significance was considered as *p* < 0.05 with a 95% level of confidence level (CI: 95%).

## Results

During the involved years 1057 medical students were studying at the four universities. Out of them 691 participated in mandatory or in elective (University of Szeged) family medicine lecture courses in the given period, who had the opportunity to participate in this research. Out of 691 students, who studied face-to-face family medicine from December 2019 to April 2020 in the Hungarian universities, 465 completed our questionnaire. The overall response rate was 67.3% (*N* = 465/691). The response rate was 86.8% (*n* = 145/167) in Debrecen, 23% (*n* = 38/165) in Pécs, 63% (*n* = 131/208) in Budapest and 73.3% (*n* = 151/206) in Szeged. Socio-demographic characteristics of the sample are presented in Table 1. Only 5% of the respondents (*N* = 23/462) plan to work as a family doctor in the future, 72% (*N* = 333/462) of them have other speciality preferences and 23% (*N* = 106/462) have not chosen their preferred speciality yet.

**Table 1:** Sample characteristics

Variable	Valid (N)	N (%)
Age [mean ± SD]	465	23.5 + − 2.1 years
Women	464	288 (62.1)
At least one parent with higher education degree	465	365 (79.0)
Being a physician’s child	465	85 (18.3)
Family or friends working in family medicine	462	121 (26.2)
Family or friends working in the preferred speciality	458	81 (17.7)
Comes from…	457	
The capital city		85 (18.6)
A big city		160 (35.0)
A small town		141 (30.8)
A rural area		71 (15.5)
University of	465	
Debrecen		145 (31.2)
Pécs		38 (8.2)
Budapest		131 (28.2)
Szeged		151 (32.5)
Year	465	
Fourth		213 (45.8)
Fifth		252 (54.2)

**Fig. 1:**
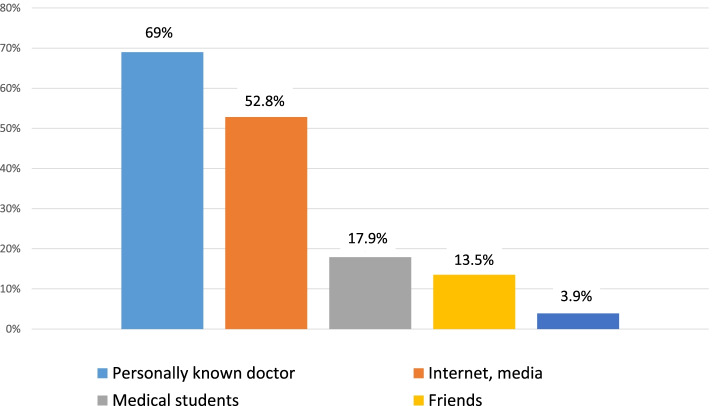
Source of information about possible future income

The vast majority of the students (91.9%; *N* = 421/458) had already thought about their future income and 47.5% (*N* = 218/459) had inquired about the exact data. The information sources of the medical students are presented in Fig. 1.

**Fig. 2:**
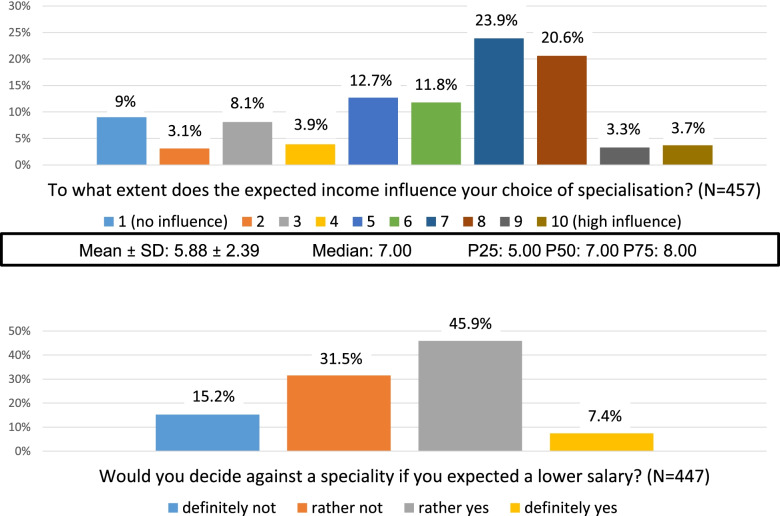
Possible effect of expected income on career choice

On a 10-point Likert scale (1 = ‘no influence’, 10 = ‘very big influence’) 76% (*N* = 347/457) answered that the expected future income has a considerable (≥5) influence on their career choice (Fig. 2). More than half of the students (*N* = 238/447) would decide against a speciality with a lower expected salary.

**Fig. 3:**
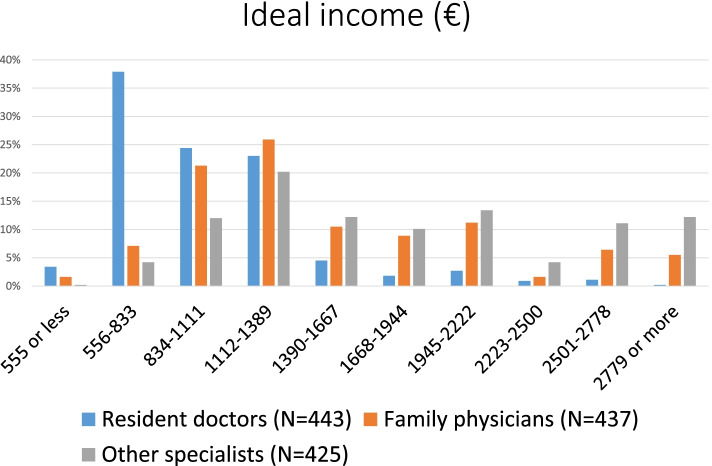
Ideal monthly incomes according to the students

Students indicated their income estimates in Hungarian forint. We used the 2020 April central rate (1 EUR = 360 HUF) to represent the data in Euros. The mean of the ideal resident, family physician and other specialist monthly income was reported to be €1154 ± 648 (P25: 833; P75: 1389), €1696 ± 904 (P25: 1111; P75: 1944) and €2174 ± 1594 (P25: 1389; P75: 2500). More than 85% of the respondents (*N* = 378/443) reported that the ideal income for a resident doctor should be between €556–1389 and 66.6% of the respondents (*N* = 291/437) said that the ideal income for a family physician should be between €834-2222. More than 95% of the respondents (*N* = 405/425) said that the ideal income for a non-family physician specialist doctor should be at least €834 (Fig. 3).

The means of the estimated incomes for resident doctors were between €631–650 depending on the location and chosen speciality. The mean of the estimated monthly income for a family physician was €1140 in rural and €1122 in urban settings; for other specialists €1081 and €1166, respectively. Students from the University of Debrecen estimated the highest income almost in all categories (except for urban specialists’ income) (Table 2).

**Table 2:** Estimated incomes (€)

	Mean + − SD	P25	P75
**Rural FM residents**	639 + − 233	486	778
**Urban FM residents**	631 + − 216	500	694
**Rural family physicians**	1140 + − 790	743	1389
**Urban family physicians**	1122 + − 532	833	1389
**Other rural residents**	633 + − 241	500	722
**Other urban residents**	650 + − 277	500	743
**Other rural specialists**	1081 + − 432	833	1250
**Other urban specialists**	1166 + − 539	833	1389

Only the minority of participants were “very certain” (1.1–8.1%) or at least “rather certain” (18–34.8%) in their estimations (Table 3).

**Table 3:** Certainty of salary estimates

	Very uncertain (%)	Rather uncertain (%)	Rather certain (%)	Very certain (%)
**Rural FM residents (*N*** **= 429)**	24	45	27.3	3.7
**Urban FM residents (*N*** **= 431)**	21.8	38.1	34.8	5.3
**Rural family physicians (*N*** **= 415)**	30.6	48.2	19	1.9
**Urban family physicians (*N*** **= 416)**	30	46.6	20.9	2.4
**Other rural residents (*N*** **= 376)**	28.7	39.1	26.1	6.1
**Other urban residents (*N*** **= 383)**	26.6	36.3	29	8.1
**Other rural specialists (*N*** **= 366)**	34.2	46.7	18	1.1
**Other urban specialists (*N*** **= 368)**	33.4	44	20.4	2.2

Students reported that the ideal income of a non-family physician specialist should be significantly higher than that of a family doctor (Table 4).

**Table 4:** The comparison of the estimated, ideal and real incomes (€) (Mean + −SD)

	Estimated	Ideal	Real
**Rural FM residents**	639 + −233	1154 + − 648	470
**Urban FM residents**	631 + −216	1154 + − 648	470
**Rural family physicians**	1140 + −790	1696 + − 904	1000
**Urban family physicians**	1122 + −532	1696 + − 904	1000
**Other rural residents**	633 + −241	1154 + − 648	470–490
**Other urban residents**	650 + −277	1154 + − 648	470–490
**Other rural specialists**	1081 + −432	2174 + − 1594	836–1170
**Other urban specialists**	1166 + −539	2174 + − 1594	836–1170

**Fig. 4:**
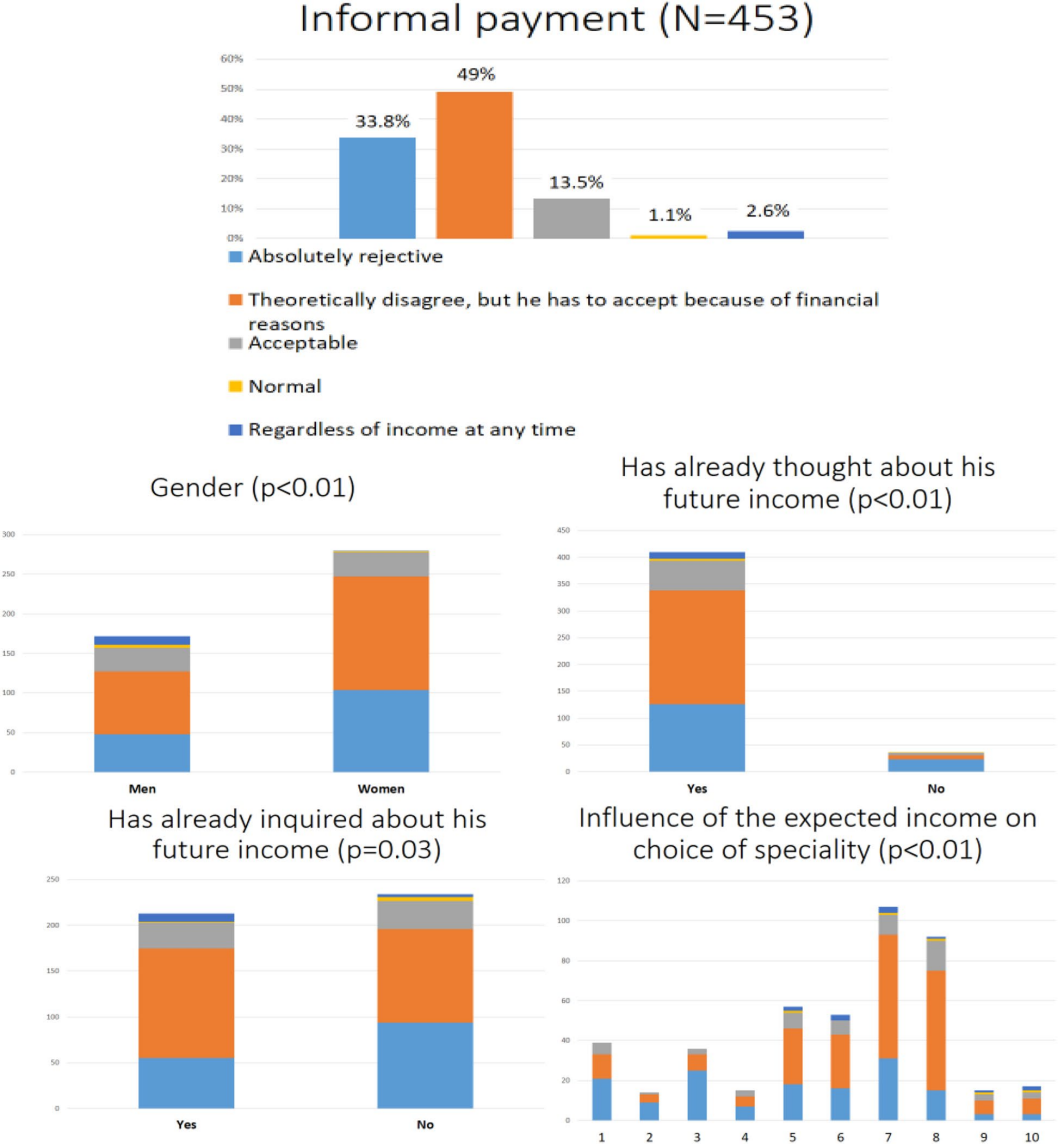
Students’ attitude towards informal payment

More than four-fifths of the respondents (*N* = 375/453) theoretically reject informal payment (Fig. 4).

## Discussion

In our study we found that expected salary plays an important role for medical students in their career choice. Most of them have already thought about it and report that the expected salary could highly influence their choice of specialisation. On the other hand, only less than half of the students have exact information in this area. Students’ information is mostly acquired from informal channels, such as doctors, other medical students or friends, and the media has a big impact as well. The university lectures provide an excellent opportunity for high quality professional training but do not create sufficient opportunities to get to know the working and living conditions associated with each speciality. Despite the information acquired through many different channels, or, rather due to that, most of the students are uncertain about the expected salaries. The importance of expected salary and uncertainty in the estimation in our study are in line with other international studies. In a recent German study, more than half of the students stated that earning opportunities are important in career choice and approximately 90% were uncertain in salary estimation. Naimer et al. in 2018 found that Israeli medical students do not have appropriate information about general practitioners’ salaries either [13, 15].

In all three categories (resident, family physician and other specialist), the estimated salaries are close to reality; however, for residents, the estimated salary is higher than the actual one. The cause of this result could be that in Hungary there are many scholarships for residents and young doctors, which could mean an extra income of €270–550 above the official salary every month. Nevertheless, this result was surprising compared to our previous study where 84% of the students underestimated the expected salaries [16]. In another one of our previous studies, most family medicine residents also underestimated expected salaries [14]. Based on these findings, we can state that Hungarian medical students are becoming more and more informed about their future earning opportunities. German medical students also significantly underestimated earning opportunities [13]. We did not find significant differences between urban and rural estimated or ideal incomes. Ideal salaries significantly exceed the estimated ones in every category. The fact that the students reported that the ideal income of a non-family physician specialist should be significantly higher than that of a family doctor may be in line with the lower prestige of family medicine among medical students [17].

Every year, women are increasingly represented in medicine, so gender differences are important to address in human resources. Eith et al. (2006) found that in Hungary fifth year women medical students want to be engaged in their medical profession but family plans are at least as important to them [18]. In our study, gender seems to play an important role in salary estimation. Men estimated higher salaries in almost every category and they were more confident in their estimates. Our findings are in line with a study carried out among surgery residents where women expected lower starting and ideal salaries [19]. A previous study in Hungary stated that among first year medical students “lifestyle and income” is more important in speciality choice for men [20]. According to international surveys, these expectations are close to reality because male physicians usually have higher salaries than their female colleagues [21, 22]. Students from the University of Debrecen estimated the highest income in all categories. This correlation was a novel finding because we know that undergraduate education has a significant role in career choice but its effect on salary expectations is not yet well-known [23–25]. Preferred speciality and motivation to work in rural settings or abroad did not influence the estimations significantly.

Attitudes of doctors and medical students towards informal payment is a key question if we want to shed light on this phenomenon of the healthcare system. In our study, we found it to be somewhat controversial. In theory, more than eight out of ten students reject it. However, two thirds of the students will accept it if the system will not change, although with different motivations. What it means is that about half of the students are influenceable by rules or circumstances. In our previous study, we found that in 2014–2015 19.7% of family physicians and 38.3% of family medicine residents were absolutely rejective of informal payment, 47 and 37.8% reported that their acceptance depends on the situation; mostly on the financial status of the patient [14]. Szinapszis Market Research and Consulting Ltd. regularly provides representative data about informal payment in Hungary. In their research in 2009 10%, in 2013 19%, in 2017 33% were absolutely rejective of informal payment. In 2009 81%, in 2013 78%, in 2017 61% accepted it in their daily work depending on the circumstances [26–28]. Based on these results we can see a positive tendency in doctors’ attitudes, but it is not enough to stop this harmful practice. Patients’ attitudes are at least as important. Baji et al. (2013) found that 47.3% of the respondents thought “Informal CASH payments to physicians and medical staff are similar to corruption.” and 54% answered that” Cash or gifts in kind, given informally to physicians and medical staff, should be eradicated.” 51.7% stated “If I have serious problems with my health, I will be ready to pay as much as I have in order to get better medical services.” From these results it can be seen, that patients do not like informal payment; however, it is a very deeply rooted phenomenon in Hungarian society [11]. We found significant correlations between acceptance of informal payment and aspects in connection with the importance of income. This finding indirectly confirm our hypothesis that if physicians would have higher income the problem of informal payments would be solved. Gender is also an influencing factor; males more often accept informal payment. The reason for this difference is ambiguous, the role of the hidden curriculum and other factors outside education, like social gender roles, may be considered [29]. Human resource recruitment in the healthcare system and informal payment are not only medical questions but affect society as a whole. Therefore it is crucial to handle them a scientific basis without any political or emotional influence.

### Strengths and limitations

This is the first study, which examined the effect of estimated future income on career choice among medical students at all of the four Hungarian medical universities. It provides current and relevant data in the topic of informal payment, which is a burning problem of the Hungarian healthcare system. The sample size and acceptable response rate allowed us to draw general conclusions. As a limitation, we have to mention that participants involved in our study included medical students from different stages of their medical education. Fourth and fifth year students may have different experiences and perceptions. The reason for this selection criteria was that we involved medical students, who were having family medicine lecture courses and the universities have different curricula. Due to the COVID-19 pandemic Hungarian universities transitioned to online education; therefore, we reached a lower response rate at the University of Pécs, which can limit the comparison of results from different universities. The cross-sectional study as study design is also a limiting factor. Cross-sectional data cannot be used to infer causality and we are not able to evaluate whether the perceptions and motivations persist in graduates. Only few of the medical students are interested in family medicine as a future specialty, so we could not describe the special characteristics of this group.

## Conclusions

In our study the students state that earning opportunities have a big impact on career choice and specialisation. Medical students in Hungary made a good estimate of their future income; however, they were not confident and mostly obtained their information from unofficial sources. This uncertainty may influence their career choice and result in the rejection of an otherwise attractive speciality. Ideal incomes given by students are in all cases significantly higher than their estimated expected incomes. This is a sad, but realistic view at the moment, which also makes it difficult to ensure adequate human resource recruitment. Most students reject informal payment theoretically. This attitude may be used as an excellent opportunity to eradicate this harmful phenomenon but further action is needed to succeed.

Further studies should examine specialisation and detect other influencing factors and changes of students’ perceptions and motivations with longitudinal examinations. A rise in medical salaries is essential to ensure the necessary amount of professionals in the healthcare system. Furthermore, providing accessible, accurate and clear information about earning opportunities is funamental. This is the common responsibility of the government, the media, medical organisations and medical education.

## Data Availability

The datasets used and/or analysed during the current study are available from the corresponding author on reasonable request.

## Abbreviations

ANOVA: Analysis of variance
CI: Confidence level
€, EUR: Euro
FM: Family medicine
GP: General practitioner
HUF: Hungarian forint
PPP: Purchasing power parity
SD: Standard deviations
